# Evaluation of saliva glutathione, glutathione peroxidase, and malondialdehyde levels in head-neck radiotherapy patients

**DOI:** 10.3906/sag-2006-84

**Published:** 2021-04-30

**Authors:** Gözde DERİNDAĞ, Hayati Murat AKGÜL, Ahmet KIZILTUNÇ, Halil İbrahim ÖZKAN, Hilal KIZILTUNÇ ÖZMEN, Nilgün AKGÜL

**Affiliations:** 1 Department of Oral and Maxillofacial Radiology, Faculty of Dentistry, Pamukkale University, Denizli Turkey; 2 Department of Biochemistry, Faculty of Medicine, Atatürk University, Erzurum Turkey; 3 Department of Radiation Oncology, Faculty of Medicine, Atatürk University, Erzurum Turkey; 4 Anesthesiology Clinical Research Office, Atatürk University, Erzurum Turkey; 5 Department of Restorative Dentistry, Faculty of Dentistry, Pamukkale University, Denizli Turkey

**Keywords:** Glutathione, glutathione peroxidase, head and neck neoplasms, malondialdehyde, radiotherapy, saliva

## Abstract

**Background/aim:**

It is believed that radiotherapy has important effects on oxidant/antioxidant systems. Oxidative stress occurs when the balance between oxidant formation and antioxidant defense is disrupted in favor of oxidants. The aim of this study was to determine the biochemical changes in saliva pre- and postradiotherapy in head-neck radiotherapy patients and to find out the effects of radiation on glutathione (GSH), glutathione peroxidase (GSH-Px), and malondialdehyde (MDA) levels in saliva.

**Materials and methods:**

This study included 16 patients undergoing head-neck radiotherapy in Atatürk University Research Hospital. The levels of GSH, GSH-Px, and MDA were measured in saliva samples taken from the patients pre- and postradiotherapy. The same biochemical parameters were also measured in saliva samples from 30 healthy individuals who did not undergo head-neck radiotherapy. The data obtained were analyzed using the paired t-test and the Mann–Whitney U test.

**Results:**

When the levels of GSH (P > 0.05), GSH-Px (P > 0.05), and MDA (P < 0.05) in saliva were compared pre- and postradiotherapy in the patient group, the only significant increase was detected in the MDA level postradiotherapy. When the pre- and postradiotherapy levels of saliva GSH (P < 0.01, P < 0.001, respectively), GSH-Px (P > 0.05, P < 0.05, respectively), and MDA (P < 0.01, P < 0.001, respectively) were compared with those of the control group, it was revealed that the GSH level was significantly lower and the MDA level was significantly higher in both pre- and postradiotherapy compared to the control group. Also, only the postradiotherapy saliva GSH-Px level was found to be significantly lower than the control group.

**Conclusion:**

These findings show that the changes in saliva GSH, GSH-Px, and MDA levels in patients with head-neck malignity intensified due to radiation.

## 1. Introduction

Head-neck cancers correspond to approximately 3%–5% of all cancer types and radiotherapy plays an important role in the treatment of these cancers [1–3]. Radiation treatment is based on reactive oxygen species (ROS) toxicity and can inflict damage in macromolecules such as DNA, RNA, microRNA, and proteins through multiple mechanisms like lipid peroxidation and enzyme oxidation [4–8].

Antioxidant defense systems are available to keep ROS formation under control and prevent the harmful effects of these molecules. Antioxidants protect normal cells against the undesirable effects of radiotherapy through various enzymatic systems [glutathione peroxidase (GSH-Px), glutathione reductase, glutathione S-transferase, superoxide dismutase, and catalase] and nonenzymatic systems [glutathione (GSH), melatonin, vitamins A, E and C, selenium, uric and ascorbic acid, beta carotene, and alpha-tocopherol] [6,9–12]. It has been suggested that antioxidant systems play a protective role in reducing oxidative damage [13]. GSH is an important intracellular antioxidant and plays an important role in neutralizing harmful compounds (particularly hydrogen peroxide), reducing oxidative stress, and activating important antioxidants [14,15]. GSH-Px is an important antioxidant that provides intracellular protection in phagocytic cells [4]. A decrease in GSH-Px activity leads to an accumulation of hydrogen peroxide (H2O2) and cellular damage. MDA, an indicator of cellular damage, is considered a biological marker of both oxidative stress and lipid peroxidation [4,7,14,16].

In the literature, parameters such as GSH, GSH-Px, and MDA have generally been investigated in different fluids such as blood, serum, and plasma in cancer patients. However, there are few studies on head and neck radiotherapy. It is thought that there might be changes in saliva quality, certain enzymes, biochemical compounds, and oxidant/antioxidant balance in patients undergoing head-neck radiotherapy. The aim of this study was to determine the biochemical changes in saliva pre- and postradiotherapy in head-neck radiotherapy patients and to determine the effect of radiation on GSH, GSH-Px, and MDA levels in saliva.

## 2. Materials and methods

### 2.1. Study design 

The study team planned to investigate the levels of GSH, GSH-Px, and MDA in saliva samples taken from head-neck radiotherapy patients who applied to the Atatürk University Research Hospital for treatment as well as from healthy individuals. After the plans were made, saliva samples were collected from healthy individuals and the patients pre- and postradiotherapy. The data obtained at the end of the biochemical analysis were compared statistically. 

### 2.2. Patients 

Patients who were to undergo head-neck radiotherapy at the Department of Radiation Oncology, the Faculty of Medicine at Atatürk University were referred to the Department of Oral and Maxillofacial Radiology, the Faculty of Dentistry at Atatürk University to remove focal infections and improve oral hygiene before treatment. Of these patients, 16 individuals who had not undergone cancer treatment (radiotherapy/chemotherapy) before, who did not have any systemic diseases (diabetes, Sjogren’s syndrome, Mikulicz disease, sarcoidosis, HIV disease, etc.) that might cause changes in saliva parameters, and who were not previously operated on their salivary glands were included in the study as the patient group. Among the patients diagnosed with head-neck cancers, eight (50%)received radiotherapy for laryngeal cancer, three (18.75%) for lymphoma, and one (6.25%) each for non-Hodgkin lymphoma, nasopharyngeal cancer, lower lip cancer, maxillary sinus tumor, and mucoepidermoid carcinoma. In the head-neck radiotherapy applied to these patients, the total fraction number was 13 to 36 days, the daily dose of radiation was 1.8 to 3 gray (Gy), and the total dose of radiation was 30 to 70 Gy. Furthermore, chemotherapy was not applied to any individuals.

Moreover, 30 individuals who applied to the Department of Oral and Maxillofacial Radiology and who had not undergone radiotherapy or did not have any periodontal and systemic diseases that might cause changes in saliva parameters were included in the study as the control group.

### 2.3. Saliva collection 

All the individuals in the patient and control groups were asked to rinse their mouths with water in a calm environment in the morning times. Then, unstimulated saliva samples were collected from them in 1.5 mL Eppendorf tubes. The saliva samples were obtained from the individuals in the patient group preradiotherapy and at the end of the second month after the start of radiotherapy. All the Eppendorf tubes were kept refrigerated at −80 °C.

### 2.4. Laboratory analysis 

The GSH, GSH-Px, and MDA levels of all the saliva samples were analyzed in the Biochemistry Laboratories at Atatürk University Research Hospital via the Enzyme-Linked Immunosorbent Assay (ELISA) method using commercial kits [ELISA Kit for glutathione (GSH), Catalog No. CEA294Ge, Wuhan USCN Business Co., Ltd., Houston, TX, USA; ELISA Kit for glutathione peroxidase 1 (GPX1), Catalog No. SEA295Hu, Wuhan USCN Business Co., Ltd.; TBARS Assay Kit for malondialdehyde (MDA), Catalog No. 10009055, Cayman Chemical, Ann Arbor, MI, USA].

### 2.5. Statistical analysis 

The data obtained at the end of biochemical analyses were transferred to the SPSS software (IBM SPSS 25.0, IBM Corp., Armonk, NY, USA). While the paired t-test was used to compare the pre- and postradiotherapy measurements in the patient group, the Mann–Whitney U test was used to compare the measurements of the patient group pre- and postradiotherapy and those of the control group.

## 3. Results

In our study, 14 out of 16 individuals in the patient group were male (87.5%), while two were female (12.5%). The mean age of the patient group was 50.68 ± 13.51 years old. In the control group, 18 out of 30 individuals were male (60%) and 12 were female (40%) and the mean age was 41.66 ± 16.44 years old. There was no statistically significant difference between genders in the control and patient groups (P > 0.05).

When the levels of GSH, GSH-Px, and MDA in saliva were compared pre- and postradiotherapy in the patient group, while the saliva GSH and GSH-Px levels decreased after radiotherapy, an increase was detected in the saliva MDA level as shown in Table 1. However, it was found that only the difference in the MDA level was statistically significant (t = 2.551, P < 0.05).

**Table 1:** Statistical comparisons of glutathione, glutathione peroxidase, and malondialdehyde levels pre- and postradiotherapy in the patient group.

	n	Preradiotherapy	Postradiotherapy	P
		mean ± sd	mean ± sd	
Glutathione (µg/mL)	16	0.885 ± 0.379	0.727 ± 0.385	0.228a
Glutathione peroxidase (ng/mL)	16	67.047 ± 53.833	62.391 ± 53.253	0.808a
Malondialdehyde (µM)	16	47.953 ± 13.398	62.171 ± 19.063	0.022b

aPaired t-test, P > 0.05; bPaired t-test, P < 0.05.

When the pre- and postradiotherapy levels of saliva GSH (0.885 ± 0.379 µg/mL and 0.727 ± 0.385 µg/mL, respectively), GSH-Px (67.047 ± 53.833 ng/mL and 62.391 ± 53.253 ng/mL, respectively), and MDA (47.953 ± 13.398 µM and 62.171 ± 19.063 µM, respectively) were compared with the control group, statistically significant differences were detected in the levels of GSH (P < 0.01 and P < 0.001, respectively) and MDA (P < 0.01 and P < 0.001, respectively). Although the pre- and postradiotherapy mean saliva GSH-Px levels (P > 0.05 and P < 0.05, respectively) were lower than the control group, a statistically significant difference was only found in the postradiotherapy measurements as shown in Tables 2 and 3. 

**Table 2:** Statistical comparison of glutathione, glutathione peroxidase, and malondialdehyde levels preradiotherapy with the control group.

	Control group		Preradiotherapy		P
	n	mean ± sd	n	mean ± sd	
Glutathione (µg/mL)	30	1.805 ± 1.370	16	0.885 ± 0.379	0.002a
Glutathione peroxidase (ng/mL)	30	75.750 ± 25.820	16	67.047 ± 53.833	0.068b
Malondialdehyde (µM)	30	34.344 ± 19.827	16	47.953 ± 13.398	0.003a

aMann–Whitney U test, P < 0.01;bMann–Whitney U test, P > 0.05.

**Table 3:** Statistical comparison of glutathione, glutathione peroxidase, and malondialdehyde levels postradiotherapy with the control group.

	Control group		Postradiotherapy		P
	n	mean±sd	n	mean±sd	
Glutathione (µg/mL)	30	1.805 ± 1.370	16	0.727 ± 0.385	0.000a
Glutathione peroxidase (ng/mL)	30	75.750 ± 25.820	16	62.391 ± 53.253	0.025b
Malondialdehyde (µM)	30	34.344 ± 19.827	16	62.171 ± 19.063	0.000a

aMann–Whitney U test, P < 0.001; bMann–Whitney U test, P < 0.05.

## 4. Discussion 

Numerous studies have been conducted in patients undergoing radiotherapy to reveal changes caused by radiation in the human body and to minimize the damages caused by radiation. In particular, the effect of radiotherapy on salivary glands has recently increased in importance and efforts have been made to protect salivary glands in head-neck radiotherapy as much as possible. Radiotherapy not only causes functional loss in salivary glands, but also creates changes in the flow rate of saliva and its amount, viscosity, pH level, buffering capacity, electrolytes, the levels of immunoglobulin and some enzymes, and oxidant/antioxidant balance [1,17,18].

So far, various studies have investigated the relationship of several enzymes, vitamins, trace elements, and oxidant/antioxidant balance in saliva with systemic and oral diseases [19,20]such as diabetes [21,22], acute coronary syndrome [7], oral lichen planus [23,24], recurrent aphthous ulcers [11,12,22,25,26], oral carcinomas [10,14], and periodontal diseases [4,7,19,25–30].However, there are few studies in the literature on oxidant/antioxidant balance in patients undergoing head-neck radiotherapy. Current studies are either restricted to certain radicals or have only investigated levels in different fluids such as blood serum or plasma. However, saliva is a much more easily obtainable fluid than blood and includes certain parameters as to oxidative stress, which is regarded as a biomarker of oral premalignant/malignant pathologies [31–33].

It has been shown that ROS damages cell components such as lipid, protein, and DNA, and plays an important role in carcinogenesis and mutagenesis [9,31]. Normally, the oxidant/antioxidant level in the body is in balance. Nevertheless, oxidative stress occurs as a result of imbalance occurring due to the increase of ROS production or inadequate antioxidant systems [7,9,15]. Generally, antioxidant and MDA levels are tested as biochemical markers of this situation [34,35].

The effects of ionized radiation used in radiotherapy on oxidant/antioxidant systems are still under discussion. Khalil Arjmandi et al. [6] investigated the levels of MDA, various antioxidants, and total antioxidants in blood samples taken pre- and postradiotherapy in patients with breast cancer and Demirci et al. [9] in patients with cervical cancer. While the former studyfound that there was a statistically significant increase only in the MDA level, the latter did not observe any variations in the MDA level. Moreover, in the latter study, the GSH and GSH-Px levels in both pre- and postradiotherapy were found to be lower compared to the control group, while the superoxide dismutase level was higher.

In head-neck malignancy patients, the effects of oral antioxidant supplementation on the levels of MDA and on certain antioxidants have been the subject of many studies [8,10]. On the one hand, Shariff et al. [8] revealed a statistically significant decrease in MDA levels postradiotherapy in patients supported with oral antioxidants, while there was a remarkable increase in the group that was not supported with oral antioxidants. On the other hand, Gupta et al. [10] detected an increase in the MDA levels postradiotherapy in both groups. However, this increase was found to be statistically significant only in the group that was not supported with antioxidants.

In our study, although there was a decrease in saliva GSH and GSH-Px levels postradiotherapy compared to preradiotherapy measurements, no statistically significant difference was detected, and a significant increase was observed only in saliva MDA level postradiotherapy (P < 0.05). The decreasing levels of GSH and GSH-Px postradiotherapy are associated with the effect of ionized radiation and significant increase in the MDA level is considered the most as the most important indicator of lipid peroxidation and oxidative stress. This increase in the MDA level might have arisen from oxidative stress not being sufficiently blocked by antioxidants due to decreasing levels of GSH and GSH-Px.

Oxidative stress resulting from an individual’s disturbance in their oxidant/antioxidant balance causes some damage in protein and lipid metabolism and plays an important role in the pathogenesis, development, progress, and prognosis of various cancer lesions [9,12,14,15,21,30,32,35–38]. Moreover, MDA, the final product of lipid peroxidation and a biomarker of oxidative stress, is involved in DNA damage, mutagenesis, and carcinogenesis [4,7,9,12,14,16,39]. When Shetty et al. [16] and Kaur et al. [32] compared the saliva MDA levels of patients at risk of malignancy and oral squamous cell carcinoma with healthy individuals, they found that the MDA levels were higher in both patient groups. In their study carried out on patients with premalignant and malignant lesions and healthy individuals, Babiuch et al. [15] found the GSH level to be lower in patient groups and they associated this with a counteracting of oxidative stress. Unlike many researchers, Fu et al. [13], found GSH-Px levels in patients with carcinoma to be significantly higher than in the control group.

In our study, the mean saliva GSH level pre- and postradiotherapy was found to be lower than in the control group (P < 0.01 and P < 0.001, respectively), while the mean saliva MDA levels were higher (P < 0.01 and P < 0.001, respectively). Although the mean saliva GSH-Px levels were found to be lower than in the control group, a statistically significant difference was found only in the postradiotherapy measurements. These differences between healthy individuals and patients with head-neck cancers can be attributed to the development mechanism of cancer lesions and tissue damage. Metabolic and mitochondrial dysfunction and frequent genetic mutations in cancer cells increase the production of ROS considerably, which leads to increased protein and lipid peroxidation [40]. Accordingly, it was reported in many cancer cases that there was a remarkable increase in serum MDA concentration [8,16]. In this study, the high saliva MDA levels in patients with head-neck cancers compared to healthy individuals support the opinion that MDA may be an indicator of DNA damage, mutagenesis, and carcinogenesis in cancer patients.

As a limitation of our study, long-term follow-up of patients (after 4 months) could not be performed due to time limitations. This follow-up of patients should be done to determine the changes and complications that may occur in the long-term postradiotherapy. In addition, the number of patients in the study remained limited due to both the time limitation and the capacity of the oncology unit where patients are referred to. Another limitation of our study was that the mean age of the individuals included in the control group was lower than that of the patient group.

In conclusion, we can say that the changes in saliva GSH, GSH-Px, and MDA levels in patients with head-neck malignancies are more intensified due to radiation. These results will help us better understand the effects of radiation on biochemical structure of saliva. Though it is known that some biochemical changes take place in saliva due to radiation, this issue has not been studied much in patients undergoing head-neck radiotherapy. Moreover, it will be an advantage, both in terms of costs and collecting samples, to favor the use of saliva over commonly used blood tissue.

## Informed consent

All the procedures followed were in accordance with the ethical standards of the responsible committee on human experimentation (institutional and national) and with the Helsinki Declaration of 1975, as revised in 2008. Informed consent was obtained from all patients for being included in the study. The Ethics Committee of the Faculty of Dentistry, Atatürk University, approved this study (Decision number: 54).
